# Analysis of non-human primate models for evaluating prion disease therapeutic efficacy

**DOI:** 10.1371/journal.ppat.1010728

**Published:** 2022-08-22

**Authors:** Meredith A. Mortberg, Eric Vallabh Minikel, Sonia M. Vallabh

**Affiliations:** 1 Stanley Center for Psychiatric Research, Broad Institute of MIT and Harvard, Cambridge, Massachusetts, United States of America; 2 McCance Center for Brain Health, Massachusetts General Hospital, Boston, Massachusetts, United States of America; 3 Department of Neurology, Massachusetts General Hospital and Harvard Medical School, Boston, Massachusetts, United States of America; 4 Prion Alliance, Cambridge, Massachusetts, United States of America; Deutsches Zentrum fur Neurodegenerative Erkrankungen, GERMANY

## Abstract

Prion disease is a fatal neurodegenerative disease caused by the conformational corruption of the prion protein (PrP), encoded by the prion protein gene (*PRNP*). While no disease-modifying therapy is currently available, genetic and pharmacological proofs of concept support development of therapies that lower PrP levels in the brain. In light of proposals for clinical testing of such drugs in presymptomatic individuals at risk for genetic prion disease, extensive nonclinical data are likely to be required, with extra attention paid to choice of animal models. Uniquely, the entire prion disease process can be faithfully modeled through transmission of human prions to non-human primates (NHPs), raising the question of whether NHP models should be used to assess therapeutic efficacy. Here we systematically aggregate data from N = 883 prion-inoculated animals spanning six decades of research studies. Using this dataset, we assess prion strain, route of administration, endpoint, and passage number to characterize the relationship of tested models to currently prevalent human subtypes of prion disease. We analyze the incubation times observed across diverse models and perform power calculations to assess the practicability of testing prion disease therapeutic efficacy in NHPs. We find that while some models may theoretically be able to support therapeutic efficacy studies, pilot studies would be required to confirm incubation time and attack rate before pivotal studies could be designed, cumulatively requiring several years. The models with the shortest and most tightly distributed incubation times are those with smaller brains and weaker homology to humans. Our findings indicate that it would be challenging to conduct efficacy studies in NHPs in a paradigm that honors the potential advantages of NHPs over other available models, on a timeframe that would not risk unduly delaying patient access to promising drug candidates.

## Author summary

Prion disease is an untreatable, rapidly fatal neurodegenerative disease. Recent therapeutic advances raise the question of which kinds of animal studies can best support drug development in prion disease. Uniquely, prions can be transmitted to and faithfully modeled in many mammals including non-human primates (NHPs), but the possibility of testing new prion disease drugs in prion-infected NHPs has never been formally evaluated. We therefore aggregated a comprehensive dataset of prion NHP studies from the literature and analyzed this dataset to determine whether, and under what conditions, this model could realistically be used to assess the efficacy of an experimental therapeutic. We find that while there is strong precedent for modeling prion disease in NHPs, a well-powered therapeutic study would be difficult to achieve on a realistic timeline due to the years-long timeframe of such studies and the lack of a well-established, predictable experimental system. We further find that the most tractable NHP models are not the most comparable to humans in terms of either brain size or prion protein gene homology, potentially reducing their utility. Finally, we suggest alternative study designs for gathering relevant animal data that may run a lower risk of delaying patient access to promising potential therapies.

## Introduction

Prion disease is a rapidly fatal neurodegenerative disease of humans and other mammals. The pathogenic mechanism pivots on the conformational corruption of a host-encoded protein, the native prion protein or PrP, into a misfolded conformer, or prion, capable of corrupting other PrP molecules and killing neurons [1]. Uniquely, prion disease can arise in three ways. Sporadic cases (~85%) appear to occur spontaneously, genetic cases (15%) trace to protein-coding variants in the prion protein gene, *PRNP* in humans [2], and acquired cases (<1%), made famous by the kuru and variant Creutzfeldt-Jakob disease (CJD) epidemics, can develop following iatrogenic exposure or consumption of prion-contaminated tissue [3]. The PrP dependence of all prion disease, regardless of etiology or even species, has long nominated the therapeutic hypothesis of PrP reduction [4], and antisense oligonucleotides (ASOs) against the prion protein RNA now provide pharmacological proof of concept for this treatment strategy [5]. This progress motivates an assessment of available model systems in which to test PrP-lowering therapies.

The prion field benefits from unusually faithful animal models. Direct inoculation of animals with prion-infected brain homogenate induces the full prion disease process in which a clinically silent incubation period gives rise to characteristic symptoms, histopathology, and biochemical features, followed by terminal illness [6]. The inoculation paradigm has replicated across a range of mammalian systems, unified by key disease hallmarks and a fatal disease endpoint, but differing in time course and attack rate according to experimental parameters including inoculation route, prion strain [7], species barrier [8], and the PrP gene dosage of the host [9,10]. Over decades, prions have been bioassayed not only in a wide range of wild-type and transgenic rodent models, but in dozens of other mammals including cervids and non-human primates (NHPs) [11]. Despite this panoply of models, most studies have relied on intracerebral inoculation of mice with a well-characterized mouse-adapted prion strain, leveraging this system’s predictable time to disease [6].

Given the rapid clinical progression of prion disease following symptom onset and ASO treatment data in mice suggesting an outsize benefit to early treatment [5], it has been proposed that PrP-lowering agents could be tested clinically in presymptomatic individuals at known risk for genetic prion disease, with a goal of delaying or preventing onset [12]. Such a clinical path could involve the FDA’s Accelerated Approval program, in which a biomarker deemed “reasonably likely to predict clinical benefit” serves as the basis for provisional approval of a new drug. Because provisional approval could thereby precede direct observation of symptomatic benefit in humans, this strategy would likely demand unusually strong supporting data from animal models. The FDA’s “Animal Rule,” while designed for therapies unable to be tested in humans at all, and thus not directly applicable here, provides some insight into how regulators’ expectations for animal studies are adjusted when human efficacy studies are not feasible [13].

The prospect of an unconventional clinical strategy draws special attention to the question of whether efficacy studies of such drugs in NHPs would be feasible or advantageous. Unlike other non-transgenic models, a number of NHP species have been shown susceptible to human prion strains on direct passage from human tissue. Other theoretical advantages could include a *PRNP* sequence relatively closer to the human gene sequence, which might permit testing of a human DNA or RNA-targeting therapy in a non-transgenic animal, and a larger brain size better suited to simulating drug delivery to the human brain. The likelihood of meeting these interests would have to be balanced against concerns about achieving adequate power to reach a meaningful clinical endpoint in a large, onerous model; ensuring that variables such as prion strain and transmission route remain faithful to clinically relevant disease paradigms; and ensuring that the length of such a study would not unnecessarily delay access to human treatments.

In order to evaluate the prospects for efficacy studies in NHPs and assess how the above interests and tradeoffs might be balanced, we set out to exhaustively catalog and analyze reported NHP models. We began with a systematic literature search to identify published articles containing original data following prion-infected NHPs to disease endpoints. We then aggregated and manually curated a dataset of individual animal cohorts and analyzed this dataset in order to i) determine how key experimental parameters in these models relate to prevalent forms of human prion disease, ii) analyze incubation times in these models and identify potential paradigms for efficacy studies, and iii) perform power calculations and assess the practicality and tradeoffs of various models.

## Methods

### Search strategy

To ensure a comprehensive and reproducible search, the following search strategy was adopted (Table A in S1 Tables). Initial searches were conducted using the PubMed online database, between 2020-04-03 and 2020-12-22, with no date range imposed upon results. The search terms “non-human primates,” “prions,” “inoculation,” “infected,” “Creutzfeldt-Jakob,” and “cynomolgus,” were used in combination. The initial results were supplemented by manual searches for the authors “Brown,” “Gajdusek,” “Marsh” and “Ono” to ensure that all work had been captured. On June 2, 2022 we expanded our PubMed search to include any papers where the Title or Abstract matched ((creutzfeldt-jakob OR spongiform OR kuru OR prion) AND (primate OR monkey OR macaque OR lemur OR chimpanzee OR gibbon OR tamarin OR primates OR monkeys OR macaques OR lemurs OR chimpanzees OR gibbons OR tamarins)), that were in English and not reviews. Citations of relevant reviews [14–16] were also screened. Titles and abstracts were reviewed for relevance, and only those containing primary data following prion-inoculated non-human primates to endpoint were included. Finally, manual follow-up was performed where reference lists in the identified reports suggested additional relevant titles.

**Fig 1:**
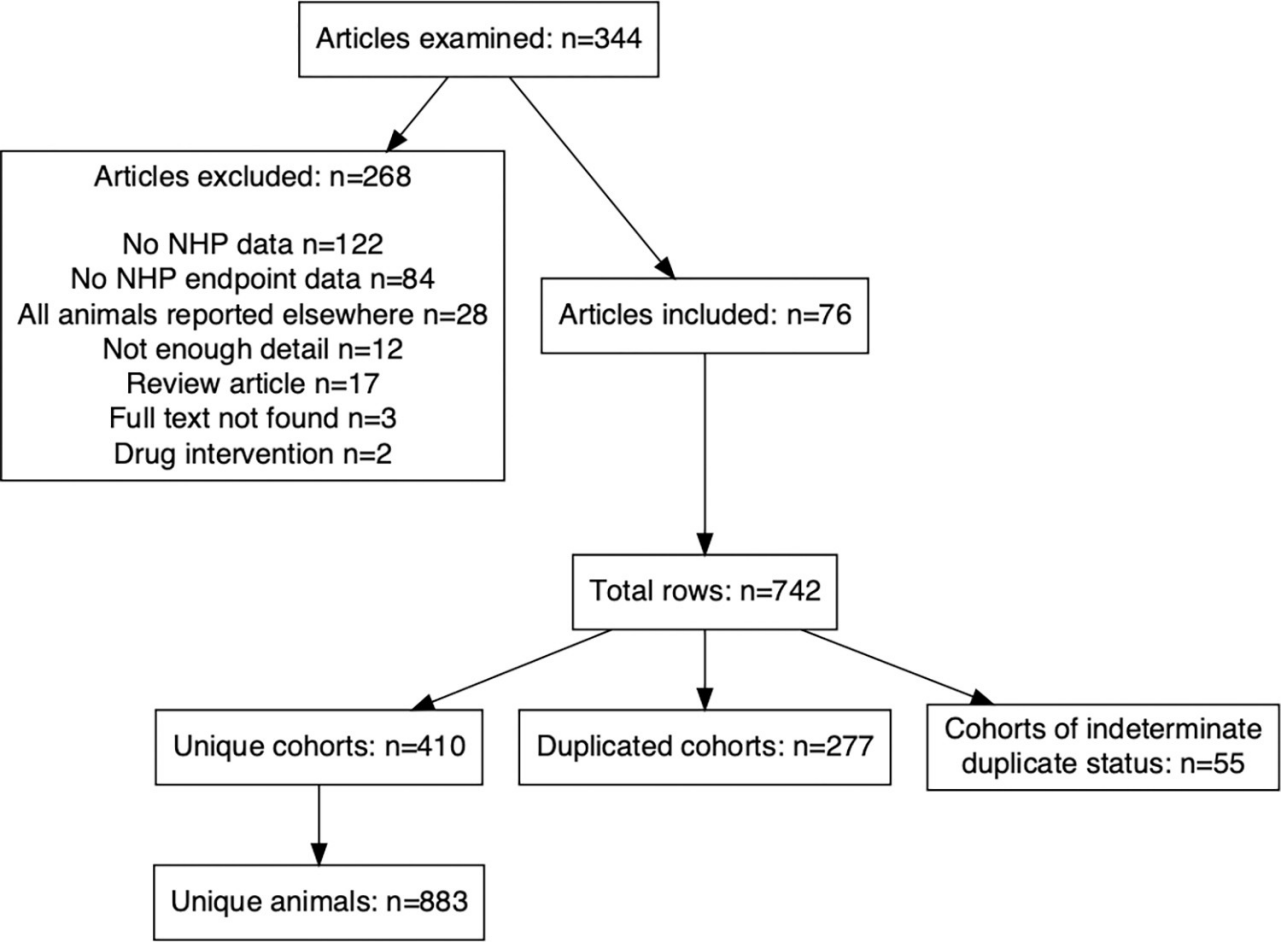
Schematic of the search strategy used to identify relevant articles.

Of 344 titles and abstracts reviewed (Fig 1), we excluded studies lacking any NHP data at all (N = 122), lacking prion endpoints in NHPs (N = 84), describing animals all reported elsewhere (N = 28), lacking sufficient detail to determine outcomes for individual animals (N = 12), review articles (N = 17), or abstracts for which we were unable to obtain full text (N = 3) (Table A in S1 Tables). We also excluded studies evaluating drug efficacy in primates (N = 2): one [17] was a conference abstract lacking experimental details, never subsequently published; the other [18] reported treatment of prion-infected cynomolgus macaques with a novel small molecule compound, but provided no characterization of this animal model to justify that the two animals were sufficient to statistically power a conclusion regarding efficacy.

Within the 76 included articles, one row was created for each unique report of an endpoint in a cohort of NHPs. Unique cohorts were defined as cohorts of animals of the same species, receiving the same prion inoculum by the same inoculation route. Many individual articles contained multiple unique cohorts, resulting in an initial count of N = 742 rows when all reported cohorts were included. However, many NHP cohorts were the subject of multiple reports spanning years or decades, reflecting either multiple experimental endpoints (e.g. histology, symptom onset, and terminal illness) and/or published updates of experiments in progress. The rows were next manually de-duplicated with the goal of including any individual animal only once. This exercise identified both duplicated cohorts and cohorts for which insufficient details exist in the literature to determine whether or not they were elsewhere reported. Both were excluded, for a final count of N = 410 unique cohorts comprising N = 883 unique animals (Fig 1). The full dataset and species list are available as Tables B and C in S1 Tables.

### Power calculations

Power calculation assumptions are enumerated under Results. For each scenario, we bootstrapped N = 1,000 iterations and power was calculated as the percentage of those iterations in which a *P* value less than 0.05 was obtained. In each iteration, survival of untreated animals was sampled from a normal distribution with the reported mean and standard deviation, while survival of treated animals was sampled from a normal distribution with 1.5 times the reported mean and 1.5 times the reported standard deviation. For the "best case scenarios", all animals were assumed to reach endpoint; for the "other scenarios", a proportion (1-p) of animals were randomly censored, where p is the reported attack rate. Survival of treated and untreated animals was then compared using a two-sided log-rank test. For each iteration, the survival time of the longest-lived animal was also recorded. The expected study duration for each scenario was calculated as the average survival time of that longest-lived animal, across the 1,000 iterations.

### Homology analysis

Sequences for the *PRNP* gene in each species, from transcription start to stop including intronic and untranslated regions, were exported from UCSC Genome Browser, except for spider monkey (*Ateles geoffroyi*), which was obtained from GenBank (PVHS01010010.1). Spider monkey, rhesus, and cynomolgus sequences, which are on the minus strand, were reverse complemented. The sequences were pairwise aligned to human *PRNP* using EMBOSS Needle [19] with default parameters. Paired alignments were trimmed to remove any extraneous sequence context. Overall percent identity was calculated as the percent of human bases aligned as matches in the NHP species. The human gene was then tiled to generate every possible 20-mer, and if all 20 bases aligned as matches, the 20-mer was considered to have perfect identity. For protein analysis, DNA sequences were translated using ExPASy [20] and open reading frames were aligned pairwise using EMBOSS Needle [19]. Residues aligned to human codons 23 through 230 (mature protein, exclusive of signal peptide and GPI signal) were considered. Insertions or deletions of a whole or partial octapeptide repeat were counted as just one mismatch.

### Statistical analysis and data availability

**Fig 2:**
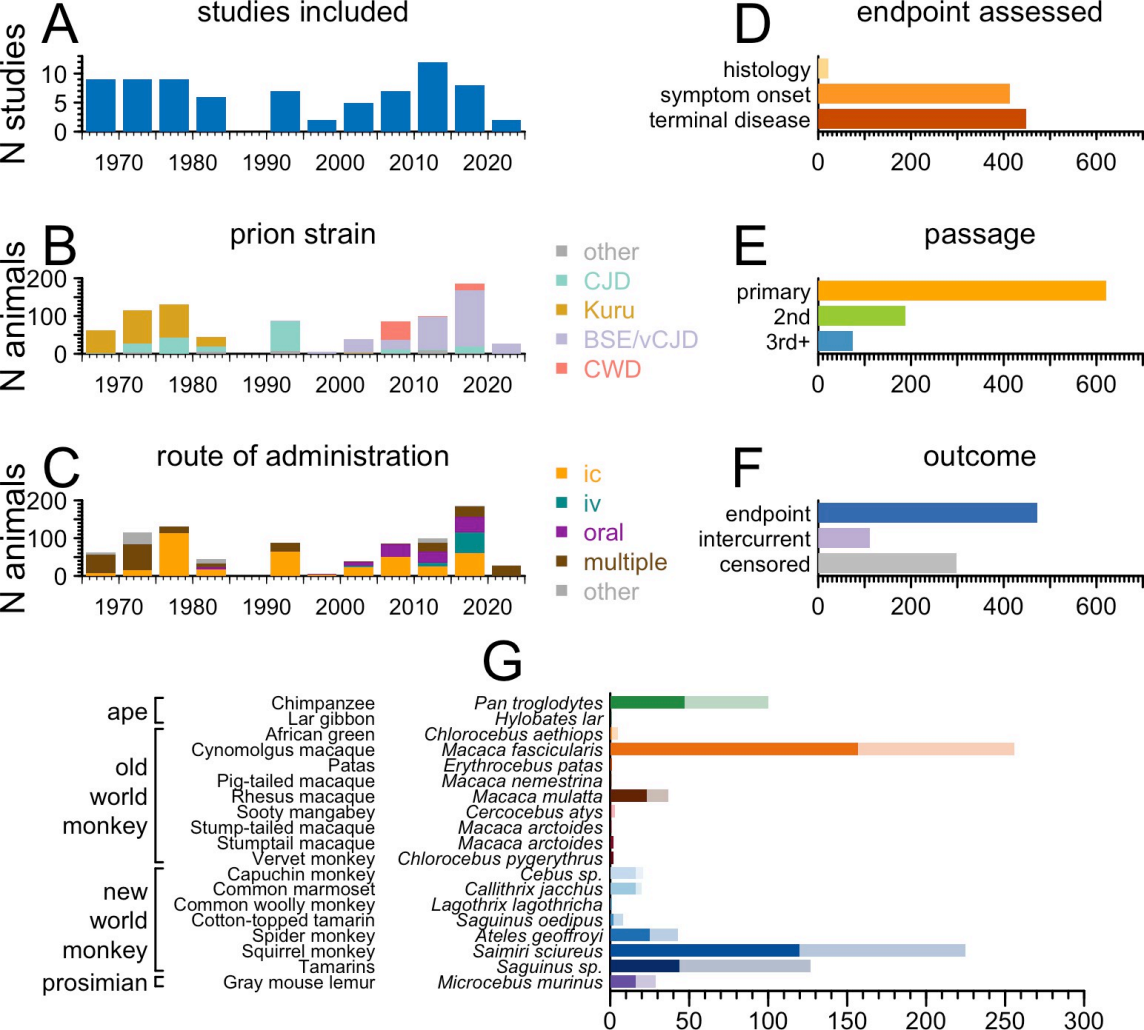
Overview of aggregated dataset of prion NHP experiments. Graphical summary of A) studies included in our analysis by year; B) prion strains studied by year; C) routes of prion administration employed, by year; and D) study endpoints, by number of NHPs. E) Passage number of the prion inoculum used, by number of NHPs. Primary refers to direct human brain isolates. Second and third+ passage refer to inocula originating from human brain tissue, that have been inoculated into NHPs, then harvested and re-inoculated into subsequent NHPs. F) The number of NHPs reaching the study’s endpoint, lost to intercurrent illness, and censored at the time of study completion. G) The number of prion-inoculated NHPs reported in the prion literature, by species. Dark bars represent animals that reached endpoint, while light bars show animals that were lost to intercurrent illness or censored.

**Fig 3:**
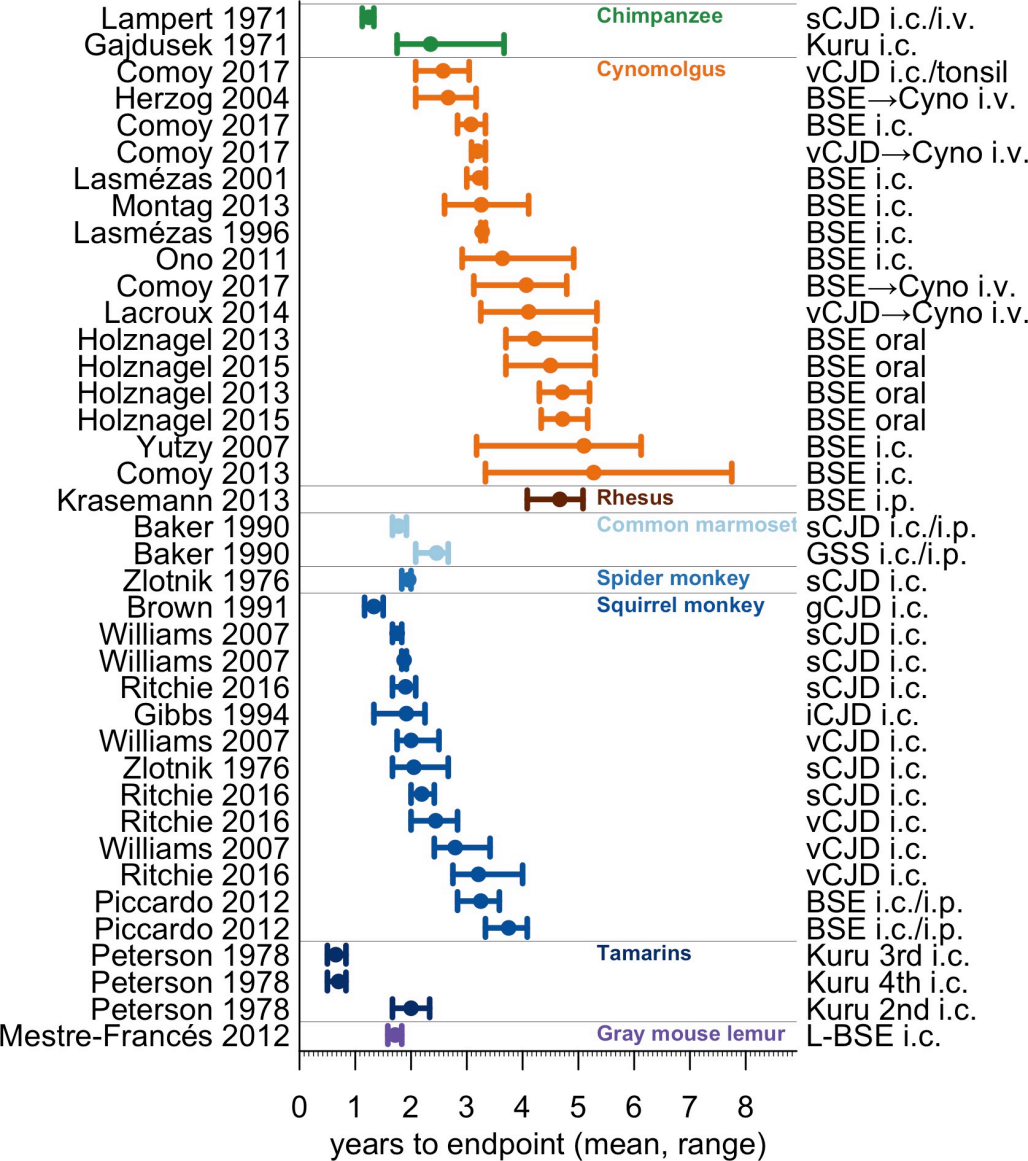
Duration of prion NHP studies. Cohorts for which it is possible to estimate the distribution of survival times were defined as those meeting all of the following criteria: i) containing at least N = 3 animals total, ii) with at least N = 3 reaching endpoint, iii) where all animals either reached endpoint or died of intercurrent illness, meaning none were censored at study termination, iv) where the endpoint studied was either terminal disease or symptom onset (as opposed to strictly histological outcomes), and v) for which the mean and standard deviation of survival time, or data sufficient to calculate such, were provided by the authors. The mean time to endpoint per cohort (dots), and range (bars), are shown alongside NHP species, strain and inoculation method.

All analyses utilized custom scripts in R 4.0.4. Statistics in Figs 1–3 are descriptive (N, mean, standard deviation, range) and are indicated in figure legends. Statistical tests and methods used in Table 1 are described under Power Calculations and Homology Analysis above. The curated dataset and source code sufficient to reproduce all analyses herein is available in a public git repository: https://github.com/ericminikel/nhp_models.

**Table 1:** Statistical power for efficacy studies in NHP models.

species	strain / RoA	study	survival time (mean±sd, months)	expected duration (months)	power	study	attack rate	survival time (mean±sd, months)	expected duration (months)	power	permitted	prevalent strain	brain size (% human)	mature protein % identity	transcript overall % identity	% 20 bp runs identity
Chimpanzee	sCJD i.c.	[31]	14.9±1.0	24	100%	[32]	24/29	17.0±7.0*	39	34%	n	y	29%	99.0%	98.5%	76.0%
Cynomolgus macaque	vCJD i.c.	[33]	30.9±4.8	56	98%						y	n	5%	95.7%	97.5%	33.8%
Rhesus macaque	BSE i.p.	[34]	56.0±6.0	95	100%						y	y	7%	95.7%	93.8%	31.3%
Common marmoset	sCJD i.c.	[35]	21.3±1.5	35	100%	[35]	3/3**	14.6±3.0	28	89%	y	y	0.6%	95.7%	90.4%	14.3%
Spider monkey	sCJD i.c.	[36]	23.5±0.8	37	100%	[32]	30/31	31.0±8.0	62	74%	y	n	8%	96.2%	90.0%	16.9%
Squirrel monkey	gCJD i.c.	[37]	16.0±2.0	28	100%	[37]	6/7	19.9±2.4	34	99%	y	y	2%	95.2%	89.0%	15.0%
	sCJD i.c.	[38]	21.0±0.8	33	100%	[32]	196/211	25.0±5.0	47	86%						
Tamarins†	Kuru→Tamarin i.c.‡	[39]	8.4±1.4	15	96%	[39]	3/3	7.8±2.0	16	75%	†	n	0.6%	95.2%	88.3%	14.2%
Gray mouse lemur	L-BSE i.c.	[40]	20.6±1.6	34	100%					34%	y	n	0.1%	93.3%	70.5%	2.4%

**(i): Best case scenarios from the literature.** Studies from the prion NHP literature representing the most rapid model for the indicated combination of species, route of administration, and strain. Assuming 6 NHPs and a therapeutic that extends survival by 50%, estimates are given for mean time to endpoint, expected duration of study (time until the last animal reaches endpoint), and power. **(ii): Other scenarios with available data.** Where available, other reported studies using the same species, strain, and inoculation route in at least N = 3 NHPs are shown for comparison, along with estimates for mean time to endpoint, duration and power. *For sCJD i.c. in chimpanzees, the attack rate of 24/29 is limited to the animals included in the mean ± sd incubation time statistics provided by Brown et al [32]; animals with longer incubation times up to 75 months are excluded. **In a separate cohort inoculated with the same inoculum as the best-case scenario, 2 animals were sacrificed for planned tissue analyses; the incubation times from the remaining 3 are shown. **(iii): Other considerations.** For each paradigm, potential motivations for conducting an efficacy study in NHPs are evaluated. “Permitted” refers to whether the species is currently available for research in the United States. “Prevalent strain” refers to current clinical relevance of the prion strain. “Brain size” is calculated based on reported mass [41–46]. “Mature protein % identity” refers to the amino acid sequence of the mature protein, see Methods; “Transcript overall % identity” refers to percent sequence identify of each species’ full *PRNP* gene compared to the human *PRNP* gene. “% 20 bp runs identity” refers to the percent of twenty base-pair runs within each species’ *PRNP* gene that are identical to the human *PRNP* gene. †Historical studies used multiple species of tamarins (*S*. *oedipus*, *S*. *fuscicollis*, *S*. *nigricollis*, and *S*. *labiatus*) interchangeably, one of which (*S*. *Oedipus*) is now endangered [47]. *PRNP* sequences were not found for any of these species, so homology statistics were calculated using the *S*. *imperator* reference genome). ‡Best-case scenario shown is from 4^th^ passage of kuru into tamarins, alternative scenario is from 3^rd^ passage.

## Results

Our systematic literature search (Methods) identified N = 76 publications reporting original data regarding prion disease endpoints in NHPs, totaling N = 410 distinct animal cohorts and N = 883 unambiguously unique individual animals (Fig 1). The temporal distribution of studies included in our analysis conformed to previous descriptions of two historical waves of primate research in the prion field [15] (Fig 2A). The first wave, in the 1970s and 80s, corresponds to large scale inoculations performed largely at the National Institutes of Health, which have been deeply recounted elsewhere [21]. When divided by prion strain, kuru emerges as the major research interest of first wave, with more recent studies focused on transmission of bovine spongiform encephalopathy (BSE) and chronic wasting disease (CWD) (Fig 2B). Notably, considering that more than 99% of prion disease cases diagnosed today are sporadic or genetic, a minority of experimental primate inoculations have used a prion subtype currently affecting human patients; the kuru and BSE/vCJD epidemics are no longer major public health threats, and despite conflicting results regarding zoonotic potential assessed in animal models, CWD is not known to have transmitted to humans [22–26].

Today, intracerebral (IC) prion inoculation is considered the highest efficiency means of experimental transmission whether for primates [15] or rodent models [6]. However, IC has not been the dominant inoculation method for primate studies (Fig 2C). When the parameters of transmission were still being explored, a wide range of techniques were tested and many animals were co-inoculated by more than one route. Meanwhile, oral inoculation is of special interest for BSE/vCJD and CWD, given that oral transmission led to zoonosis of BSE to humans [27], and poses what is considered to be the greatest risk of zoonosis of CWD [28]. In addition, recent study of the intravenous (IV) method has been spurred by the discovery that vCJD has been transmitted via blood transfusion to four humans [29]. In total, 382/883 animals in our search were inoculated by the IC method alone.

Most animals reviewed were followed with the intention of observing a clinical endpoint of either symptoms or terminal disease (Fig 2D), following inoculation with a primary prion strain from a natural host, rather than a strain that had already undergone passage through non-human primates (Fig 2E). Notably, however, given the length and difficulty of primate studies, roughly as many animals were either censored or lost to incurrent illness as were successfully followed to endpoint (410 vs. 473, Fig 2F).

Cynomolgus macaques (*Macaca fascicularis*) were the most heavily represented primate species across studies, and squirrel monkeys (*Saimiri sciureus*) were the second-most studied (Fig 2G). Chimpanzees (*Pan troglodytes*), while also well represented historically, are effectively no longer used for prion research following decisions by the NIH to phase out funding for chimpanzee research in 2013, and the U.S. Fish and Wildlife Service designation of all chimpanzees as endangered in 2015 [30].

Broadly, NHP studies have sought to characterize prion transmission potential across diverse paradigms, spanning species, strains, and transmission routes (Fig 2). While it is clear that such studies take years, the time to endpoint varies both between and within experimental paradigms. 84% (345/410) of reported NHP cohorts have consisted of fewer than 4 animals, with 52% (215/410) of cohorts consisting of only 1 animal. If we limit our view to cohorts of at least N = 3 animals, for which it is possible to estimate the distribution of survival times (see Fig 3 legend), then many combinations of species, strain and transmission route have been tested in only one experiment. For the paradigm that has been tested the greatest number of times–intracerebral inoculation of BSE into cynomolgus macaques–it is clear that these three variables alone do not standardize time to onset (Fig 3).

For each of the eight species represented in Fig 3, we selected the potentially most tractable combination of prion strain and route of administration for further analysis to determine the characteristics of a potential therapeutic efficacy study in each paradigm (Table 1). In order to calculate statistical power for such studies, we made the following assumptions:

1. Efficacy study of a therapeutic would require at least N = 6 NHPs (N = 3 of each sex, as Animal Rule Guidance recommends equal male and female representation).
2. Animals would be followed to terminal endpoint.
3. The therapeutic intervention would convey a 50% increase in survival time.
4. The outcome would be evaluated by log-rank survival test with a two-sided alpha = 0.05 statistical threshold.
5. The study would last until the last animal reaches endpoint.

Based on these assumptions, we calculated the expected study duration and statistical power (1-β, probability of correctly rejecting the null hypothesis) for each paradigm under either i) the best-case scenario from the literature or ii) any other available reports, and tabulated these along with iii) other considerations for each model (Table 1). Other considerations (Table 1, Section iii) included whether use of the model is permitted for research, relevance of the prion strain, and brain size, as well as homology to the human prion protein and two metrics of homology to the human *PRNP* transcript. Because antisense oligonucleotide therapeutics, a modality currently in development for prion disease, are 20 base pairs long and are generally intolerant to single mismatches, we calculated not only overall percent transcript identity, but also the percent of possible 20 base pair sequences that are 100% identical to the human sequence, a proxy for the probability of a drug designed for humans happening to match each species.

The best-case scenario for each paradigm would yield >90% power with study durations ranging from just over one to eight years, though it is possible that preliminary evidence of efficacy could be gleaned sooner. However, several caveats apply. First, as suggested by Fig 3, it is not always the case that these combinations of species, strain, and inoculation route can be counted on to generate comparable results across studies. Indeed, where available, other reports in these paradigms suggest that attack rate may be lower, and/or incubation time longer, than observed in the “best case” report. The low, tightly distributed incubation time in the “best case” report for each paradigm might arise in part from luck, given the small number of NHPs in each cohort, and/or from properties of the exact brain sample inoculated, which may not still be available today.

In addition, not all motivations for performing an NHP study are satisfied by the paradigms highlighted in Table 1. Five out of eight paradigms involve either a species (chimpanzees) no longer available, or a prion strain (vCJD, BSE, or kuru) not responsible for many human prion disease cases today. Meanwhile increasing phylogenetic divergence from humans corresponds to steep drops in both NHP brain mass and *PRNP* sequence identify, particularly as measured in terms of the multi-base pair stretches of identity likely to be required to support targeting with a human-relevant genetically targeted therapy.

## Discussion

Here, we set out to assess the feasibility of conducting preclinical efficacy studies of experimental prion disease therapeutics in prion-infected NHPs. Efficacy studies in an NHP disease model are not typically gating for either human testing of, or approval of a new drug; for example, neurological ASOs recently advanced to the clinic have consistently done so based on demonstrations of efficacy in rodent disease models, complemented by pharmacokinetic, pharmacodynamic, and safety data from wild-type NHPs [48–50]. However, we nevertheless felt it was important to address this topic for prion disease, for two reasons. First, unlike for many other diseases, compelling NHP models of terminal prion disease are available. Second, because prion disease is so rapid once it strikes, preventive trials in asymptomatic carriers of pathogenic *PRNP* variants have been proposed. Trials of experimental therapeutics in individuals with no detectable disease process may require unusually strong preclinical data, prompting an evaluation of all available tools.

We found that transmission of human prions to NHPs is well established, and has been achieved independently by multiple laboratories dating back to the 1960s. Inocula representing multiple human prion strains have proven transmissible to multiple NHP species by multiple routes of inoculation. In this sense, NHP models of prion disease have been deeply explored. Many prion-inoculated cohorts of NHPs, however, consisted of only one or two animals per experimental condition, and/or exhibited incomplete attack rates or highly variable incubation times. Restricting our analysis to cohorts of three or more prion-inoculated NHPs that developed terminal illness with a full attack rate reveals a more constrained universe of available models. Of these models, several paradigms in New World monkeys have achieved complete or near-complete attack rates in two years or less, with standard deviations of a few months. It is possible that a study in one of these species could offer reasonable power to detect a therapeutic effect within a few years using a clinically relevant prion strain such as sCJD.

Nevertheless, our analysis highlights the challenges and limitations that such a study would face. Different studies have yielded incubation times of different magnitude and variability, potentially impacting study duration and statistical power (Table 1). This variability may derive from the fact most NHP experiments utilized primary passage of human prions (Figs 2E and 3), with a distinct human brain isolate serving as inoculum in each study. Unlike in mice, where serial passage has given rise to well-characterized prion strains with typical incubation times and consistent terminal titers, distinct human brain isolates could reasonably be expected to differ in transmission-relevant properties such as prion titer and precise molecular subtype. The desire to ensure that a costly therapeutic efficacy study will not be wasted, should animal endpoints prove more variable than expected, might lead prudent sponsors to first conduct a pilot study to confirm incubation time for the exact prion isolate and inoculation procedure to be employed. Such a study would add years of up-front model development effort before a therapeutic efficacy study could begin, bringing the cumulative expected timeframe of a pilot study plus subsequent pivotal efficacy study to several years (Table 1). If such studies were gating for drug approval, they might unduly delay patient access to effective drugs.

Meanwhile, it is debatable whether the New World monkeys with the most rapid incubation times observed here would honor all of the motivations for pursuing NHP studies to begin with. While their brain architecture may more closely mirror that of humans, their brain mass is a tiny fraction of that of a human brain (ranging from 0.6% to 8%), smaller than those of sheep, goats, pigs, and large dogs [46], so it is debatable whether they provide an advantage over non-NHP models in assessing drug brain distribution. Meanwhile, if efficacy studies need to be conducted with the actual human drug candidate rather than a surrogate compound [13], the greater divergence of these New World monkeys from humans [51] may pose an obstacle. In the context of a 20-base pair nucleic acid therapeutic, only about one-sixth of each species’ *PRNP* sequence is composed of 20-meric runs of identity compared to the human gene. If a drug targeting the human gene is unlikely to show cross-reactivity by chance, cross-reactivity might only be achieved if it were prioritized in drug candidate selection, potentially compromising other drug parameters. While this analysis focuses by way of example of an oligonucleotide therapeutic targeting the prion protein RNA, the broader consideration of sequence identity is likely to be a consideration for other genetically targeted drug modalities.

An alternative to the use of NHP prion infection models is to use separate models to address each question about a drug. A possible scheme is outlined in Table 2. The details will vary depending upon the therapeutic hypothesis—for example, animals overexpressing PrP may be valuable tools for rapid evaluation of some therapeutic agents, but would likely underestimate the benefit of PrP lowering due to the non-linear relationship between PrP dosage and disease tempo [10]. Considering the example of PrP lowering, it has been possible to interrogate the relationship of the therapeutic hypothesis to PrP dosage, prion strain, and disease stage in wild-type mice, and the target engagement biomarker of CSF PrP has proven responsive in rats [5,52], while potent compounds have been identified in uninfected humanized mice [53]. Per FDA guidance, new drug programs generally require pharmacology and toxicology studies in at least two species including one non-rodent [54]; drug distribution and activity in a larger brain would be evaluated as part of such a program. What additional evidence will be required to link pharmacologic PrP lowering both to a drug-responsive biomarker and to an improved disease outcome will be a matter of further discussions with regulators.

**Table 2:** Potential roles of different animal models in prion disease drug development.

Model system	Key goals
Wild-type mice infected with murine prions	• Test therapeutic hypothesis • Evaluate prion strain specificity • Assess dose-response relationship between target engagement and disease outcome • Interrogate disease stage dependence
Other small animals or transgenic mice	• Validate therapeutic hypothesis in additional species and/or against additional prion strains
Uninfected humanized mice	• Screen for most potent human-targeting compounds
Humanized mice infected with human prions	• Establish relevance of therapeutic hypothesis to human prion strains
Uninfected wild-type mice and rats	• Toxicology and pharmacology studies • Target engagement biomarker assessment
Uninfected non-human primates and/or other large animals	• Toxicology and pharmacology studies, ADME, and immunological assessments • Brain distribution studies • Target engagement biomarker validation

Overall, the distribution of drug development activities across multiple more rapid and less costly models could allow a tighter feedback loop whereby insights from one experiment help to inform a follow-on experiment, and allow a greater diversity of experimental parameters such as prion strain, drug dose, dosing regimen, and time of administration to be varied. By contrast, an efficacy study in an NHP model would require an intense investment of time and resources in a single experiment.

In summary, available data suggest that an NHP efficacy study of a prion disease therapeutic would be imaginable but daunting. The costs and benefits would need to be carefully weighed in light of both the drug type in question and the status of the drug development program to determine whether the scientific gains would outweigh the potential delay in advancing a therapeutic to human clinical trials.

## Supporting information

S1 Tables Table A: All prion NHP reports from which data were evaluated for inclusion in the present study. Table B: The full prion-infected NHP dataset used for analysis. Table C: All NHP species used in the studies evaluated for inclusion. Supplemental References.
(XLSX)

## References

[1] Prusiner SB. Prions. PNAS. 1998;95: 13363–13383. doi: 10.1073/pnas.95.23.13363 9811807 PMC33918

[2] Mead S, Lloyd S, Collinge J. Genetic Factors in Mammalian Prion Diseases. Annu Rev Genet. 2019;53: 117–147. doi: 10.1146/annurev-genet-120213-092352 31537104

[3] Will RG. Acquired prion disease: iatrogenic CJD, variant CJD, kuru. Br Med Bull. 2003;66: 255–265. doi: 10.1093/bmb/66.1.255 14522863

[4] Lledo PM, Tremblay P, DeArmond SJ, Prusiner SB, Nicoll RA. Mice deficient for prion protein exhibit normal neuronal excitability and synaptic transmission in the hippocampus. Proc Natl Acad Sci U S A. 1996;93: 2403–2407. doi: 10.1073/pnas.93.6.2403 8637886 PMC39809

[5] Minikel EV, Zhao HT, Le J, O’Moore J, Pitstick R, Graffam S, et al. Prion protein lowering is a disease-modifying therapy across prion disease stages, strains and endpoints. Nucleic Acids Res. 2020;48: 10615–10631. doi: 10.1093/nar/gkaa616 32776089 PMC7641729

[6] Watts JC, Prusiner SB. Mouse Models for Studying the Formation and Propagation of Prions. J Biol Chem. 2014;289: 19841–19849. doi: 10.1074/jbc.R114.550707 24860095 PMC4106304

[7] Bartz JC. Prion Strain Diversity. Cold Spring Harb Perspect Med. 2016;6: a024349. doi: 10.1101/cshperspect.a024349 27908925 PMC5131755

[8] Collinge J, Clarke AR. A general model of prion strains and their pathogenicity. Science. 2007;318: 930–936. doi: 10.1126/science.1138718 17991853

[9] Büeler H, Aguzzi A, Sailer A, Greiner RA, Autenried P, Aguet M, et al. Mice devoid of PrP are resistant to scrapie. Cell. 1993;73: 1339–1347. doi: 10.1016/0092-8674(93)90360-3 8100741

[10] Fischer M, Rülicke T, Raeber A, Sailer A, Moser M, Oesch B, et al. Prion protein (PrP) with amino-proximal deletions restoring susceptibility of PrP knockout mice to scrapie. EMBO J. 1996;15: 1255–1264. 8635458 PMC450028

[11] Brandner S, Jaunmuktane Z. Prion disease: experimental models and reality. Acta Neuropathol. 2017;133: 197–222. doi: 10.1007/s00401-017-1670-5 28084518 PMC5250673

[12] Vallabh SM, Minikel EV, Schreiber SL, Lander ES. Towards a treatment for genetic prion disease: trials and biomarkers. The Lancet Neurology. 2020;19: 361–368. doi: 10.1016/S1474-4422(19)30403-X 32199098

[13] U.S. Food and Drug Administration. Product Development Under the Animal Rule: Guidance for Industry. 2015 Oct. Available: https://www.fda.gov/regulatory-information/search-fda-guidance-documents/product-development-under-animal-rule

[14] Trevitt CR, Collinge J. A systematic review of prion therapeutics in experimental models. Brain. 2006;129: 2241–2265. doi: 10.1093/brain/awl150 16816391

[15] Krasemann S, Sikorska B, Liberski PP, Glatzel M. Non-human primates in prion research. Folia Neuropathol. 2012;50: 57–67. 22505364

[16] Comoy EE, Mikol J, Deslys J-P. Non-human primates in prion diseases. Cell Tissue Res. 2022. doi: 10.1007/s00441-022-03644-7 35661921

[17] Amyx H, Salazar AM, Gajdusek DC, Gibbs CJ. Chemotherapeutic Trials in Experimental Slow Virus Diseases. Neurology. 1984. p. Suppl. 1 PP102.6197678

[18] Yamaguchi K, Kamatari YO, Ono F, Shibata H, Fuse T, Elhelaly AE, et al. A designer molecular chaperone against transmissible spongiform encephalopathy slows disease progression in mice and macaques. Nat Biomed Eng. 2019;3: 206–219. doi: 10.1038/s41551-019-0349-8 30948810

[19] Madeira F, Park YM, Lee J, Buso N, Gur T, Madhusoodanan N, et al. The EMBL-EBI search and sequence analysis tools APIs in 2019. Nucleic Acids Res. 2019;47: W636–W641. doi: 10.1093/nar/gkz268 30976793 PMC6602479

[20] Gasteiger E, Gattiker A, Hoogland C, Ivanyi I, Appel RD, Bairoch A. ExPASy: The proteomics server for in-depth protein knowledge and analysis. Nucleic Acids Res. 2003;31: 3784–3788. doi: 10.1093/nar/gkg563 12824418 PMC168970

[21] Asher DM. Kuru: memories of the NIH years. Philos Trans R Soc Lond B Biol Sci. 2008;363: 3618–3625. doi: 10.1098/rstb.2008.4002 18849246 PMC2735519

[22] Kong Q, Huang S, Zou W, Vanegas D, Wang M, Wu D, et al. Chronic Wasting Disease of Elk: Transmissibility to Humans Examined by Transgenic Mouse Models. J Neurosci. 2005;25: 7944–7949. doi: 10.1523/JNEUROSCI.2467-05.2005 16135751 PMC6725448

[23] Race B, Williams K, Chesebro B. Transmission studies of chronic wasting disease to transgenic mice overexpressing human prion protein using the RT-QuIC assay. Veterinary Research. 2019;50: 6. doi: 10.1186/s13567-019-0626-2 30670087 PMC6341683

[24] Wang Z, Qin K, Camacho MV, Cali I, Yuan J, Shen P, et al. Generation of human chronic wasting disease in transgenic mice. Acta Neuropathologica Communications. 2021;9: 158. doi: 10.1186/s40478-021-01262-y 34565488 PMC8474769

[25] Hannaoui S, Zemlyankina I, Chang SC, Arifin MI, Béringue V, McKenzie D, et al. Transmission of Cervid prions to Humanized Mice Demonstrates the Zoonotic Potential of CWD. bioRxiv; 2022. p. 2022.04.19.488833. doi: 10.1101/2022.04.19.488833 PMC9468132 35996016

[26] Wadsworth JDF, Joiner S, Linehan JM, Jack K, Al-Doujaily H, Costa H, et al. Humanized Transgenic Mice Are Resistant to Chronic Wasting Disease Prions From Norwegian Reindeer and Moose. The Journal of Infectious Diseases. 2021; jiab033. doi: 10.1093/infdis/jiab033 33502474 PMC9470110

[27] Will RG, Ironside JW, Zeidler M, Cousens SN, Estibeiro K, Alperovitch A, et al. A new variant of Creutzfeldt-Jakob disease in the UK. Lancet. 1996;347: 921–925. doi: 10.1016/s0140-6736(96)91412-9 8598754

[28] Hannaoui S, Schatzl HM, Gilch S. Chronic wasting disease: Emerging prions and their potential risk. PLoS Pathog. 2017;13: e1006619. doi: 10.1371/journal.ppat.1006619 29095921 PMC5667731

[29] Urwin PJM, Mackenzie JM, Llewelyn CA, Will RG, Hewitt PE. Creutzfeldt-Jakob disease and blood transfusion: updated results of the UK Transfusion Medicine Epidemiology Review Study. Vox Sang. 2016;110: 310–316. doi: 10.1111/vox.12371 26709606

[30] Grimm D. Chimps in waiting | Science. 16 Jun 2017356: 1114–1117. 10.1126/science.356.6343.1114 28619896

[31] Lampert PW, Gajdusek DC, Gibbs CJ. Experimental spongiform encephalopathy (Creutzfeldt-Jakob disease) in chimpanzees. Electron microscopic studies. J Neuropathol Exp Neurol. 1971;30: 20–32. doi: 10.1097/00005072-197101000-00004 4925307

[32] Brown P, Gibbs CJ, Rodgers-Johnson P, Asher DM, Sulima MP, Bacote A, et al. Human spongiform encephalopathy: the National Institutes of Health series of 300 cases of experimentally transmitted disease. Ann Neurol. 1994;35: 513–529. doi: 10.1002/ana.410350504 8179297

[33] Comoy EE, Mikol J, Jaffré N, Lebon V, Levavasseur E, Streichenberger N, et al. Experimental transfusion of variant CJD-infected blood reveals previously uncharacterised prion disorder in mice and macaque. Nat Commun. 2017;8: 1268. doi: 10.1038/s41467-017-01347-0 29097653 PMC5668246

[34] Krasemann S, Mearini G, Krämer E, Wagenführ K, Schulz-Schaeffer W, Neumann M, et al. BSE-associated prion-amyloid cardiomyopathy in primates. Emerg Infect Dis. 2013;19: 985–988. doi: 10.3201/eid1906.120906 23735198 PMC3713817

[35] Baker HF, Duchen LW, Jacobs JM, Ridley RM. Spongiform encephalopathy transmitted experimentally from Creutzfeldt-Jakob and familial Gerstmann-Sträussler-Scheinker diseases. Brain. 1990;113 (Pt 6): 1891–1909. doi: 10.1093/brain/113.6.1891 2276050

[36] Zlotnik I, Grant DP, Dayan AD, Earl CJ, Illis LS, Weller RO. Further observations on the experimental transmission of Creutzfeldt-Jakob disease from man to squirrel and spider monkeys. Neuropathology and Applied Neurobiology. 1976;2: 125–130. doi: 10.1111/j.1365-2990.1976.tb00490.x

[37] Brown P, Goldfarb LG, Gibbs CJ, Gajdusek DC. The phenotypic expression of different mutations in transmissible familial Creutzfeldt-Jakob disease. Eur J Epidemiol. 1991;7: 469–476. doi: 10.1007/BF00143124 1684754

[38] Williams L, Brown P, Ironside J, Gibson S, Will R, Ritchie D, et al. Clinical, neuropathological and immunohistochemical features of sporadic and variant forms of Creutzfeldt-Jakob disease in the squirrel monkey (Saimiri sciureus). J Gen Virol. 2007;88: 688–695. doi: 10.1099/vir.0.81957-0 17251588

[39] Peterson DA, Wolfe LG, Deinhardt FW. Human spongiform encephalopathies in marmoset monkeys (Saguinus sp.). Primates Med. 1978;10: 254–260. 417327

[40] Mestre-Francés N, Nicot S, Rouland S, Biacabe A-G, Quadrio I, Perret-Liaudet A, et al. Oral transmission of L-type bovine spongiform encephalopathy in primate model. Emerg Infect Dis. 2012;18: 142–145. doi: 10.3201/eid1801.111092 22261009 PMC3310119

[41] Hartwig W, Rosenberger AL, Norconk MA, Owl MY. Relative brain size, gut size, and evolution in New World monkeys. Anat Rec (Hoboken). 2011;294: 2207–2221. doi: 10.1002/ar.21515 22042631

[42] Herndon JG, Tigges J, Klumpp SA, Anderson DC. Brain weight does not decrease with age in adult rhesus monkeys. Neurobiol Aging. 1998;19: 267–272. doi: 10.1016/s0197-4580(98)00054-2 9662002

[43] MacLean EL, Barrickman NL, Johnson EM, Wall CE. Sociality, ecology, and relative brain size in lemurs. J Hum Evol. 2009;56: 471–478. doi: 10.1016/j.jhevol.2008.12.005 19410273

[44] Svennerholm L, Boström K, Jungbjer B. Changes in weight and compositions of major membrane components of human brain during the span of adult human life of Swedes. Acta Neuropathol. 1997;94: 345–352. doi: 10.1007/s004010050717 9341935

[45] Pardo ID, Garman RH, Weber K, Bobrowski WF, Hardisty JF, Morton D. Technical guide for nervous system sampling of the cynomolgus monkey for general toxicity studies. Toxicol Pathol. 2012;40: 624–636. doi: 10.1177/0192623311436180 22317925

[46] Crile G, Quiring Daniel P. A Record of the Body Weight and Certain Organ and Gland Weights of 3690 Animals. Ohio Journal of Science. 1940;40: 219–259.

[47] Savage A, Thomas L, Feilen KL, Kidney D, Soto LH, Pearson M, et al. An Assessment of the Population of Cotton-Top Tamarins (Saguinus oedipus) and Their Habitat in Colombia. PLoS One. 2016;11: e0168324. doi: 10.1371/journal.pone.0168324 28030570 PMC5193348

[48] DeVos SL, Miller RL, Schoch KM, Holmes BB, Kebodeaux CS, Wegener AJ, et al. Tau reduction prevents neuronal loss and reverses pathological tau deposition and seeding in mice with tauopathy. Sci Transl Med. 2017;9. doi: 10.1126/scitranslmed.aag0481 28123067 PMC5792300

[49] McCampbell A, Cole T, Wegener AJ, Tomassy GS, Setnicka A, Farley BJ, et al. Antisense oligonucleotides extend survival and reverse decrement in muscle response in ALS models. J Clin Invest. 128: 3558–3567. doi: 10.1172/JCI99081 30010620 PMC6063493

[50] Tabrizi SJ, Leavitt BR, Landwehrmeyer GB, Wild EJ, Saft C, Barker RA, et al. Targeting Huntingtin Expression in Patients with Huntington’s Disease. New England Journal of Medicine. 2019 [cited 11 Jun 2019]. doi: 10.1056/NEJMoa1900907 31059641

[51] Perelman P, Johnson WE, Roos C, Seuánez HN, Horvath JE, Moreira MAM, et al. A molecular phylogeny of living primates. PLoS Genet. 2011;7: e1001342. doi: 10.1371/journal.pgen.1001342 21436896 PMC3060065

[52] Mortberg MA, Zhao HT, Reidenbach AG, Gentile JE, Kuhn E, O’Moore J, et al. Regional variability and genotypic and pharmacodynamic effects on PrP concentration in the CNS. JCI Insight. 2022;7: e156532. doi: 10.1172/jci.insight.156532 35133987 PMC8986079

[53] Freier SM, Bui H-H, Zhao H. Compounds and methods for reducing prion expression. WO2020106996A1, 2020. Available: https://patents.google.com/patent/WO2020106996A1/en

[54] U.S. Food and Drug Administration. M3(R2) Nonclinical Safety Studies for the Conduct of Human Clinical Trials and Marketing Authorization for Pharmaceuticals. Guidance for Industry. 2010. Available: https://www.fda.gov/media/71542/download 20349552

